# Clinical impact of preoperative diaphragm dysfunction on early outcomes and ventilation function in lung transplant: a single-center retrospective study

**DOI:** 10.1186/s40560-022-00614-7

**Published:** 2022-05-15

**Authors:** Sungchul Huh, Woo Hyun Cho, Dohyung Kim, Bong Soo Son, Hye Ju Yeo

**Affiliations:** 1 Department of Rehabilitation Medicine, Pusan National University Yangsan Hospital, Pusan National University School of Medicine, Yangsan, South Korea; 2 Division of Pulmonary, Allergy and Critical Care Medicine, Department of Internal Medicine, Pusan National University Yangsan Hospital, Pusan National University School of Medicine, Geumo-ro 20, Beomeo-ri, Mulgeum-eup, Yangsan-si, Gyeongsangnam-do 626-770 Republic of Korea; 3 Department of Thoracic and Cardiovascular Surgery, Pusan National University Yangsan Hospital, Pusan National University School of Medicine, Yangsan, South Korea; 4 Research Institute for Convergence of Biomedical Science and Technology, Pusan National University Yangsan Hospital, Geumo-ro 20, Beomeo-ri, Mulgeum-eup, Yangsan-si, Gyeongsangnam-do 626-770 Republic of Korea

**Keywords:** Diaphragmatic dysfunction, Lung transplantation, Electrical impedance tomography, Computed tomography, Ultrasound

## Abstract

**Background:**

Clinical impact of preoperative diaphragm dysfunction on lung transplantation has not been studied. We aimed to evaluate how preoperative diaphragm dysfunction affects clinical outcomes and ventilation function after transplantation.

**Methods:**

We retrospectively enrolled 102 patients. Ultrasound for diagnosis of diaphragm dysfunction was performed on all patients both before and after lung transplantation. The primary outcome was to compare prolonged mechanical ventilation after transplantation according to the preoperative diaphragm dysfunction. Secondary outcomes compared global inhomogeneity index and lung volume after transplantation. Multivariate regression analysis were used to evaluate the association between preoperative diaphragm dysfunction and prolonged mechanical ventilation after transplantation.

**Results:**

A total of 33 patients (32.4%) had preoperative diaphragm dysfunction, and half of them (*n* = 18) recovered their diaphragm function after transplantation. In contrast, 15 patients (45.5%) showed postoperative diaphragm dysfunction. The ratio of prolonged mechanical ventilation after transplantation was significantly higher in the preoperative diaphragm dysfunction group (*p* = 0.035). The postoperative durations of mechanical ventilation, intensive care unit and hospital stays were higher in the preoperative diaphragm dysfunction group, respectively (*p* < 0.05). In the multivariate regression analysis, preoperative diaphragm dysfunction was significantly associated with prolonged mechanical ventilation after transplantation (Odds ratio 2.79, 95% confidence interval 1.07–7.32, *p* = 0.037). As well, the preoperative diaphragm dysfunction group showed more inhomogeneous ventilation (*p* < 0.05) and lower total lung volume (*p* < 0.05) after transplantation. In addition, at 1 month and 3 months after transplantation, FVC was significantly lower in the preoperative diaphragm dysfunction group (*p* < 0.05).

**Conclusions:**

Preoperative diaphragm dysfunction was associated with prolonged mechanical ventilation after lung transplantation.

**Supplementary Information:**

The online version contains supplementary material available at 10.1186/s40560-022-00614-7.

## Background

Lung transplantation (LT) is the only treatment option to restore quality of life and overcome shortness of breath in patients with end-stage lung disease [1, 2]. The latest registry from the International Society for Heart and Lung Transplantation reports 1- and 5-year survival rates of 85% and 59% for adult LT recipients, respectively [1]. The LT survival rate is still lower than that of transplants in other organs [2]. However, there have been some improvements in LT over the past decade, including donor selection, organ preservation, perioperative management, and postoperative management and rehabilitation [1]. Among several factors that affect the survival of LT recipients, preoperative diaphragm dysfunction (DD) may be a significant factor. DD is closely related to underlying end-stage lung diseases, such as idiopathic pulmonary fibrosis [3]. In addition, various causes such as critical illness polyneuropathy, critical illness myopathy, lung hyperinflation, inflammatory conditions, drugs, prolonged ventilators, and other metabolic causes complexly affect DD [4]. In general, since the diaphragm plays a role in 80% of inspirations of pulmonary function, forced vital capacity (FVC) can be reduced by 75% in bilateral dysfunction [5]. In addition, it can cause impaired lung ventilation, which leads to atelectasis and pneumonia [6–8]. Therefore, preoperative DD can affect not only quality of life but also overall success and survival rate after LT. Several studies on the relationship between postoperative DD and clinical outcomes after LT have been reported [9, 10]. However, studies regarding preoperative DD are still lacking. It is necessary to understand the impact of preoperative DD on early outcomes and ventilation function after LT.

Electrical impedance tomography (EIT) is a radiation-free imaging modality used to assess lung aeration. It provides both static and dynamic information on the overall and regional distribution of ventilation [11]. In addition, three-dimensional computed tomography (3D-CT) volumetry is a reliable method for assessing lung volume and is even more reproducible than total lung capacity measurements using pulmonary function tests [12]. In this study, we investigated the impact of preoperative DD on ventilator use after transplantation. As well, we evaluated the difference in ventilation and lung function after LT according to preoperative DD using EIT and 3D-CT volumetry.

## Methods

### Study population

Patients who underwent preoperative diaphragm ultrasonography among double LT recipients between April 2017 and May 2021 were included in this study. We retrospectively analyzed the clinical medical records including EIT and 3D-CT volumetry. The Institutional Review Board of the local ethical committee of Pusan National University Yangsan Hospital approved the current retrospective study (IRB no. 05-2021-164). Informed consent was waived because of the retrospective nature of the study. All selected participants scored between − 1 and + 1 on the Richmond Agitation–Sedation Scale and had a negative score on the Confusion Assessment Method for Intensive Care Units. The exclusion criteria were as follows: age < 18 years, presence of massive pleural effusion, pneumothorax, pneumomediastinum, neuromuscular blocker usage within 48 h, obesity (body mass index ≥ 30 kg/m^2^), and presence of an electrical device (implantable cardioverter–defibrillator or pacemaker). The following characteristics of the participants were collected: age, sex, body mass index, diagnosis of pre-transplant lung disease, medical support prior to the LT, survival rate, ultrasonographic diaphragm assessment results, 3D-CT volumetry results, and EIT results.

### Diagnosis of DD

DD was defined as a diaphragm excursion of ≤ 10 mm on tidal breathing or the presence of paradoxical movement on ultrasound [13, 14]. Ultrasonography was performed in the supine position for pre-transplantation evaluation at the time of transplant registration. The postoperative assessment using ultrasound was performed at 3 months and 1 year after LT. Ultrasonography was performed using M-mode tracing with a 5-MHz probe for excursion and B mode with a 15-MHz transducer for thickness. The thickening fraction of the diaphragm (TFdi) was defined as the following formula: (Thickness at end-inspiration − Thickness at end-expiration)/Thickness at end-expiration × 100. A trained examiner performed the ultrasonographic examination for consistency. All measurements were collected by the same examinator (Additional file 1) [15, 16].

### Study outcomes and variables

The primary outcome was to compare prolonged mechanical ventilation after transplantation according to the preoperative diaphragm dysfunction. Secondary outcomes compared global inhomogeneity index and lung volume after transplantation using EIT and 3D-CT volumetry.

### EIT

EIT measurements were performed postoperatively on day 7 using the PulmoVista 500 (Dräger Medical GmbH, Lübeck, Germany) for those breathing spontaneously (Additional file 1). An array of 16 electrodes was placed around the chest wall in the sixth intercostal space. Ventilation distribution between the right and left lungs at both the upper and lower lung segments during tidal breathing was assessed. To quantify the tidal volume distribution, the EIT-based global inhomogeneity (GI) index was calculated, which represents an asymmetrical distribution in the lungs [17]. To reduce the influence of fluid overload and pleural effusion on EIT, we excluded patients with pulmonary congestion and pleural effusion detected on ultrasonography.

### Lung volume measurement using 3D-CT volumetry

Helical CT was performed using a 64-multidetector CT scanner (Phillips Brilliance Series; Phillips Medical Systems, Best, the Netherlands) with patients in the supine position. A CT scan was performed both 1 week and 3 months after LT. The scans were calibrated to include both lungs completely. The patients were instructed to hold their breath during deep inspiration. Each image series was assessed for proper quality and adherence to the protocol. Post-processing of the images was performed using software at an independent workstation (3D slicer) [18]. This software enables automatic segmentation of the lungs based on a threshold density of − 750 HU and a region of interest, thereby revealing 3D volume-rendered images. The trachea, up to the level of the thoracic inlet, was included in the volume measurements. Manual segmentation was additionally performed if misclassification of the digestive tract occurred. Split lung volumetric assessment was performed using manual segmentation, separating the two lungs down the midline of the trachea. The lung volumes were calculated automatically by the software as the sum of the volumes of voxels included in the segmentation.

### Spirometry

Spirometry was performed 1 month and 3 months after LT. FVC was measured in the sitting position using a spirometer (VMax 20; Viasys, San Diego, CA, USA).

### Definitions

Preoperative DD was defined as a patient who was diagnosed with DD in the pre-transplant evaluation. Postoperative DD was diagnosed by ultrasonography at 3 months after LT, including medical and surgical causes. Sustained pre-operative DD was defined as the continuation of preoperative DD at 3 months after LT. A newly developed postoperative DD was defined as a case with new developed DD at 3 months after LT. Persistent DD was defined as a patient diagnosed with DD at 1 year after LT.

Prolonged mechanical ventilation (PMV) was defined as greater than 21 days of mechanical ventilation for at least 6 h per day [19].

### Statistical analysis

All data are presented as medians and interquartile ranges or means and standard deviations for continuous variables, as appropriate. The Mann–Whitney rank-sum test (non-parametric values) or independent *t* tests (parametric values) were used to compare these variables. Categorical variables were compared using Fisher’s test or chi-squared test. A two-sided *p* value of < 0.05 was considered significant. The paired *t* test was used to compare the FVC, total lung volume, and each segment volume between 1 and 3 months in each group. Univariate regression analysis was performed to determine the clinical factors affecting prolonged mechanical ventilation after transplantation. Multivariate regression analysis was conducted on all factors with *p* < 0.10 using a logistic regression model. All statistical analyses were performed using SPSS for Windows version 26.0 (SPSS Inc., Chicago, IL).

## Results

### Baseline characteristics

**Fig. 1:**
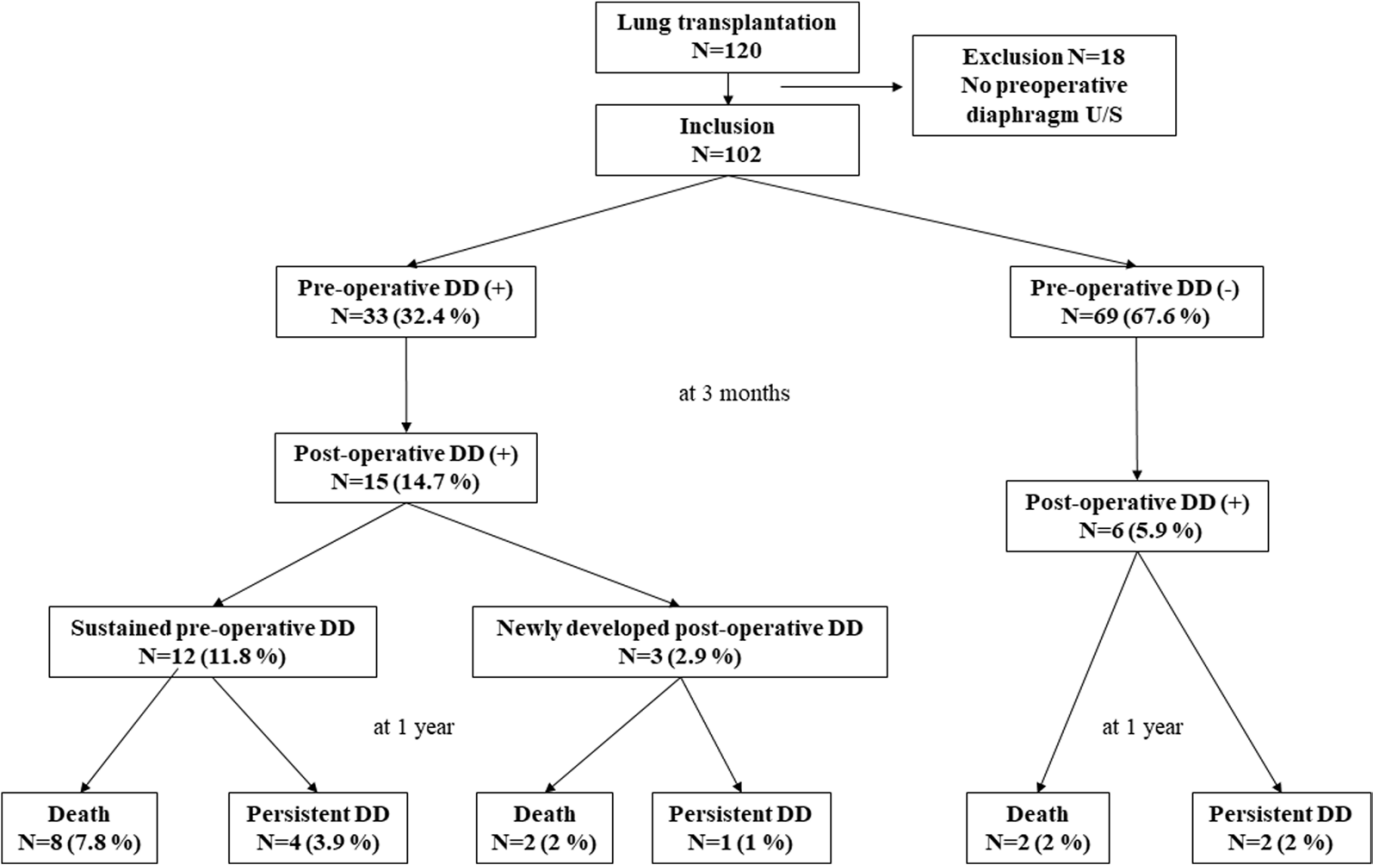
Patient enrollment. *DD* diaphragm dysfunction. In total, 32.4% of patients showed preoperative DD. Of those, 54.5% recovered diaphragm function 3 months after transplantation

During the study periods, 120 patients have received lung transplantation at our center. A total of 102 patients were included in this study except for 18 patients without preoperative diaphragm sonographic results (Fig. 1). Of these, 32.4% (33/102) had preoperative DD, and 57.6% (19/33) had a unilateral DD. DD was detected in 24 patients (72.7%) on the right side and in 23 patients (69.7%) on the left side. The baseline characteristics according to preoperative DD are presented in Table 1. The baseline characteristics were not different between the two groups except for mechanical ventilator duration before LT (11 vs. 1 day, *p* = 0.004) and right heart dysfunction (33.3% vs. 10.1%, *p* = 0.004). The median diaphragm excursion during tidal breathing was significantly lower in the DD group than that in the non-DD group (right, 0.8 vs. 2 cm, *p* < 0.001; left, 0.5 vs. 2 cm, *p* < 0.001). The median diaphragm excursion with forced breathing was also significantly lower in the DD group than that in the non-DD group (right, 1.2 vs. 3.3 cm, *p* < 0.001; left, 1.5 vs. 3.5 cm, *p* < 0.001). The TFdi of the right side and left side were also significantly lower in the DD group (right 24 vs. 100%, *p* = 0.001; left 26 vs. 96.5%, *p* = 0.006).

**Table 1:** Clinical characteristics according to pre-operative diaphragm dysfunction

	Preoperative DD (+) (*n* = 33)	Preoperative DD (−) (*n* = 69)	*p* value
Age, years	56.0 ± 12.2	57.8 ± 8.9	0.410
Male, *N* (%)	17 (51.5)	46 (66.7)	0.141
BMI, kg/m^2^	21.4 ± 5.0	21.1 ± 3.9	0.731
APACHE II	18.6 ± 7.5	16.1 ± 8.3	0.156
Diagnosis, *N* (%)			
IPF	26 (78.8)	52 (75.4)	0.503
COPD	3 (9.1)	4 (5.8)	
Bronchiectasis	0	3 (4.3)	
BO	4 (12.1)	7 (10.1)	
Others	0	3 (4.3)	
Pre-transplantation, *N* (%)			
MV	25 (75.8)	41 (59.4)	0.106
ECMO	17 (51.5)	36 (52.2)	0.950
Pre-operative MV duration†, days	11 (1–26)	1 (0–9.5)	0.004
Right heart dysfunction before transplantation, *N* (%)	11 (33.3)	7 (10.1)	0.004
Diaphragm excursion†, cm			
Tidal breathing, right	0.8 (0.3–1.3)	2 (1.5–2.5)	< 0.001
Tidal breathing, left	0.5 (0–1.0)	2 (1.8–3.2)	< 0.001
Forced breathing, right	1.2 (0.5–2.5)	3.3 (2.2–4.7)	< 0.001
Forced breathing, left	1.5 (0.5–2.7)	3.5 (2.6–4.8)	< 0.001
Thickening fraction†, (%)			
Thickening fraction, right	24 (0–93)	100 (41.5–130.5)	0.001
Thickening fraction, left	26 (12.5–102)	96.5 (50–134)	0.006
Donor factor, *N* (%)			
Size mismatch	20 (60.6)	35 (50.7)	0.349
Sex mismatch	12 (36.4)	23 (33.3)	0.763
Wedge resection	10 (30.3)	8 (11.6)	0.020
Lobar transplantation	3 (9.1)	1 (1.4)	0.063

Other data presented as means ± SD or as numbers (percentage)

*DD* diaphragm dysfunction, *BMI* body mass index, *APACHE II* Acute Physiology and Chronic Health Evaluation II, *LT* lung transplantation, *IPF* idiopathic pulmonary fibrosis, *COPD* chronic obstructive pulmonary disease, *BO* bronchiolitis obliterans, *MV* mechanical ventilator, *ECMO* extracorporeal membrane oxygenation

^†^Data are presented as medians (interquartile range)

### Clinical outcomes

The clinical outcomes of the two groups are shown in Table 2. There was no significant difference in hospital mortality between the two groups. However, the ratio of prolonged mechanical ventilation was significantly higher in the DD group (36.4% vs. 17.4%, *p* = 0.035). The duration of ventilator use in the post-transplantation period was significantly higher in the DD group (15 vs. 6.5 days, *p* = 0.003). The lengths of intensive care unit (ICU) stays and hospital stays were significantly higher in the DD group (ICU stays, 20 vs. 11.5 days, *p* = 0.016; hospital stays, 86 vs. 50.5 days, *p* = 0.001).

**Table 2:** Clinical outcomes

	Preoperative DD (+) (*n* = 33)	Preoperative DD (−) (*n* = 69)	*p* value
Post-transplantation			
Ventilator duration, days^†^	15 (7–37.5)	6.5 (3–18.0)	0.003
Prolonged mechanical ventilation (greater than 21 days after surgery) [19], *N* (%)	12 (36.4)	12 (17.4)	0.035
Length of ICU stay, days^†^	20 (13–42)	11.5 (6–21.5)	0.016
Length of hospital stay, days^†^	86 (60–133.5)	50.5 (29.3–74.8)	0.001
Hospital mortality, *N* (%)	7 (21.2)	9 (13.0)	0.289
Spirometry			
FVC at 1 month (%)	44.2 ± 18.0	53.1 ± 17.8	0.045
FVC at 3 months (%)	55.7 ± 18.7	65.2 ± 17.6	0.041
Total lung volume of 3D-CT volumetry, liter^†^			
1 week	1.7 (1.1–2.4)	2.5 (1.8–3.1)	0.002
3 months	2.0 (1.4–2.8)	3.0 (2.0–3.8)	0.018

Other data are presented as means ± SD or as numbers (percentage)

*DD* diaphragm dysfunction, *ICU* intensive care unit, *FVC* forced vital capacity, *3D-CT* three-dimensional computed tomography

^†^Data are presented as medians (interquartile range)

### Preoperative factors associated with prolonged mechanical ventilation after transplant

Univariate regression analysis results are shown in Table 3. In the multivariate regression analysis, pre-operative DD was significantly associated with prolonged mechanical ventilation after transplant (odds ratio 2.79, 95% confidence interval 1.07–7.32, *p* = 0.037).

**Table 3:** Multivariate regression analysis for prolonged mechanical ventilation after lung transplant

	Univariate		Multivariate	
	OR (95% CI)	*p*	OR (95% CI)	*p*
ECMO bridge to transplantation	2.33 (0.90–6.08)	0.083		
MV bridge to transplantation	2.51 (0.85–7.41)	0.097		
Pre-operative DD	2.71 (1.06–6.97)	0.038	2.79 (1.07–7.32)	0.037

*OR* odds ratio, *CI* confidence interval, *ECMO* extracorporeal membrane oxygenation, *MV* mechanical ventilator, *DD* diaphragm dysfunction

### Comparison of GI index values between the two groups

**Fig. 2:**
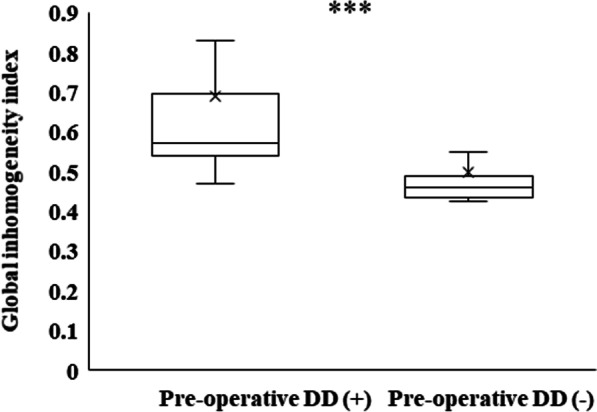
Global inhomogeneity index according to the diaphragm dysfunction. Data are presented as medians (interquartile range). DD, diaphragm dysfunction; *** *p* < 0.001. Electrical impedance tomography was performed in 58 patients 1 week after transplantation. The global inhomogeneity index was significantly higher in the DD group (0.5 vs. 0.6, *p* < 0.001)

EIT was conducted in 58 patients 1 week after LT. The median GI index value was significantly higher in the DD group (0.6 [0.5–0.7] vs. 0.5 [0.4–0.5], *p* < 0.001, Fig. 2). In other words, the inhomogeneity of ventilation was significantly higher in the DD group.

### Lung volume assessment and serial changes determined using 3D-CT volumetry and spirometry

**Fig. 3:**
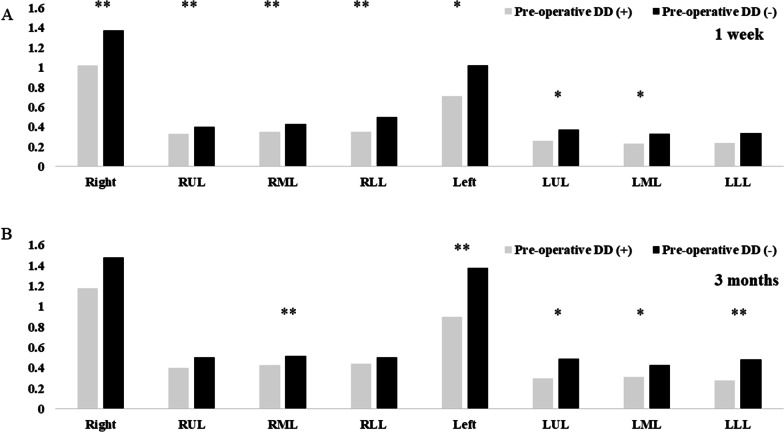
Each lung volume segment acquired using three-dimensional computed tomography volumetry 1 week and 3 months after transplant. *p < 0.05, **p < 0.01,***p < 0.001. The data show the lung volume (L) of each segment. There were significant differences in each segment’s lung volume at 1 week and 3 months between the two groups. *DD* diaphragm dysfunction

The total lung volume in 3D-CT volumetry was significantly lower in the DD group than that in the non-DD group (1 week, 1.7 vs. 2.5 L, *p* = 0.002; 3 months, 2.0 vs. 3.0 L, *p* = 0.018, Table 2). In addition, there were significant differences in each segment’s lung volume between the two groups (Fig. 3). The FVC at 1 and 3 months after LT was significantly lower in the pre-operative DD group, respectively (1 month, 44.2% vs. 53.1%, *p* = 0.045, 3 months, 55.7% vs. 65.2%, = 0.041).

**Fig. 4:**
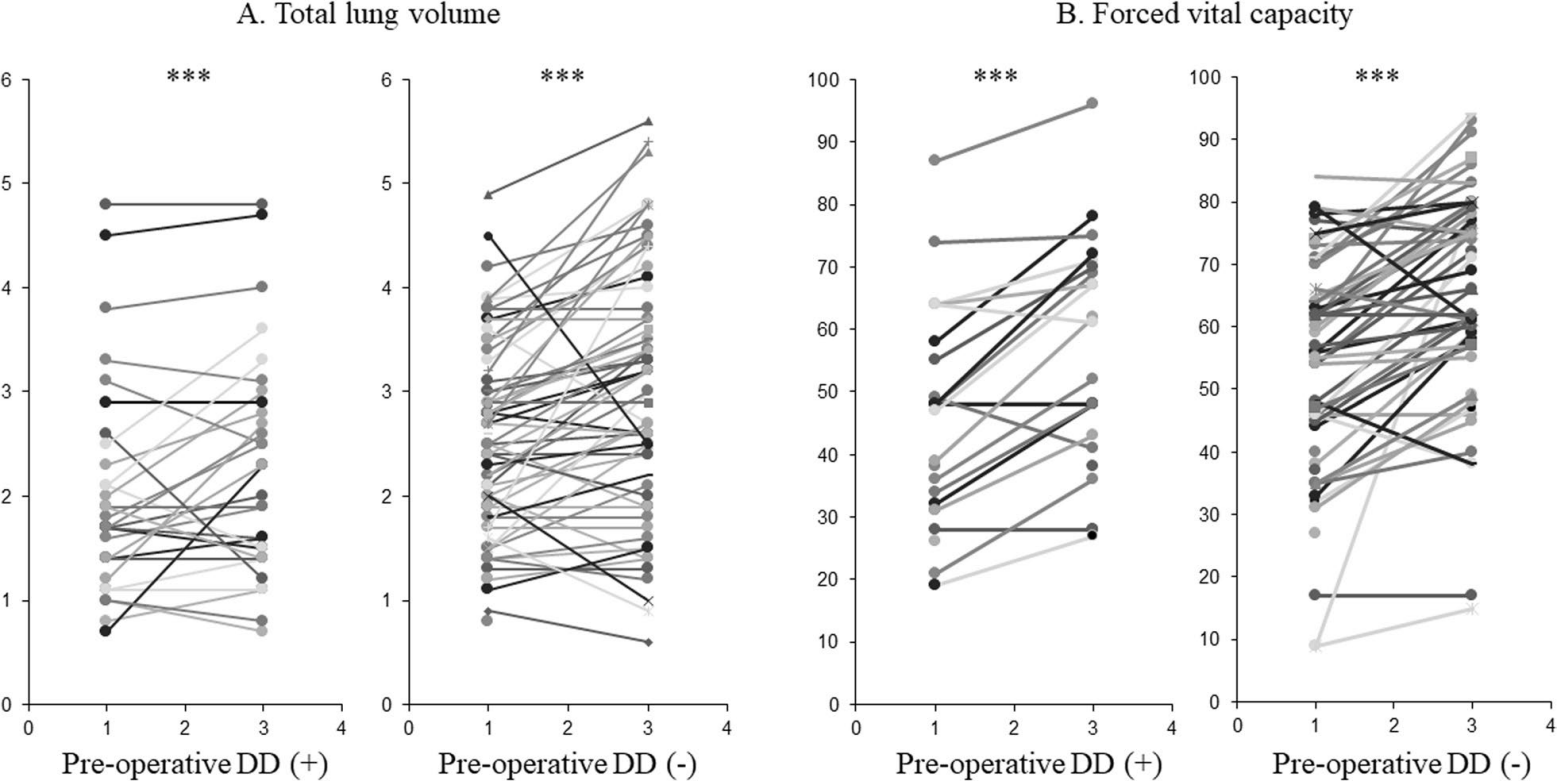
Paired *t* test for total lung volume and forced vital capacity (FVC). **A** Paired *t* test for total lung volume using three-dimensional computed tomography (3D-CT) volumetry. The total lung volume determined by 3D-CT volumetry was significantly increased from 1 week to 3 months in both groups regardless of preoperative DD (patients with preoperative DD, *p* < 0.001; patients without preoperative DD, *p* < 0.001). **B** Paired *t* test for FVC using spirometry FVC significantly increased from 1 to 3 months in both groups regardless of preoperative DD (patients with preoperative DD, *p* < 0.001; patients without preoperative DD, *p* < 0.001). ***p < 0.001. *DD* diaphragm dysfunction

In both groups, the total lung volume was significantly increased from 1 week to 3 months (DD, 1.9 vs. 2.2 L, *p* < 0.001; DD (−), 2.6 vs. 3.0 L, *p* < 0.001, Fig. 4A). The serial change in total lung volume between the two periods tended to be lower in the DD group (0.2 vs. 0.4 L, *p* = 0.074).

In both groups, the FVC was significantly increased from 1 to 3 months regardless of the presence of DD (DD, 1.7 vs. 2.1 L, *p* < 0.001; DD (−), 2.2 vs. 2.7 L, *p* < 0.001, Fig. 4B). There was no significant difference in the serial change of FVC between the two groups (0.3 vs. 0.4 L, *p* = 0.448).

### Natural course of pre-operative DD

In the group with preoperative DD, 54.5% (18/33) recovered diaphragm function at 3 months after transplantation, and the other 45.5% (15/33) showed DD at 3 months after transplantation (Fig. 1). Among those, 12 patients showed DD in the same site as before surgery, and the remaining 3 patients had newly developed DD in the other site after surgery (Additional file 1). In patients without preoperative DD, six (8.7%) showed postoperative DD. Seven patients showed persistent DD after 1 year of LT.

The clinical outcomes were significantly different between two groups according to diaphragm function at 3 months (Table 4). The mortality rate of patients with unimproved DD at 3 months was significantly higher than patients with improved DD (*p* = 0.001). Although the FVC were not different between two groups, total lung volume of 3D CT volumetry were significantly different between two groups.

**Table 4:** Clinical outcomes of patients with pre-operative diaphragm dysfunction according to diaphragm recovery at 3 months

	DD at 3M (+) (*n* = 15)	Without DD at 3M (*n* = 18)	*p* value
Post-transplantation			
Ventilator duration, days^†^	20 [10–46]	10.5 [5–32]	0.056
Length of ICU stay, days^†^	33 [15–70]	17.5 [10–26]	0.009
Length of hospital stay, days^†^	16 [76–137]	67.5 [48.8–114.8]	0.056
Hospital mortality, *N* (%)	7 (46.7)	0	0.001
Spirometry			
FVC at 1 month (%)	38.9 ± 12.7	46.8 ± 19.9	0.318
FVC at 3 months (%)	59.6 ± 15.1	54.5 ± 19.9	0.606
Total lung volume of 3D-CT volumetry, liter^†^			
1 week	1.7 [1.1–1.9]	1.9 [1.3–2.6]	0.048
3 months	1.4 [1.1–2.0]	2.5 [1.8–3.1]	0.003

Other data are presented as means ± SD or as numbers (percentage)

*M* month, *DD* diaphragm dysfunction, *ICU* intensive care unit, *FVC* forced vital capacity, *3D-CT* three-dimensional computed tomography

^†^Data are presented as medians (interquartile range)

### Regression analysis between pre-operative DD and clinical factors

In the univariate regression analysis, regarding preoperative clinical factors, ventilator duration (odds ratio [OR] 1.04, 95% confidence interval [CI] 1.01–1.07, *p* = 0.015), ICU duration (OR 1.03, 95% CI 1.01–1.05, *p* = 0.012) and right heart failure (OR 4.43, 95% CI 1.53–12.85, *p* = 0.006) were significantly associated with preoperative DD. In the multivariate regression analysis, ICU duration (OR 1.02, 95% CI 1.00–1.04, *p* = 0.018) and right heart failure (OR 4.86, 95% CI 1.56–15.15, *p* = 0.006) were significantly associated with preoperative DD.

## Discussion

The current study which aimed to evaluate how preoperative DD affects clinical outcomes after LT showed that preoperative DD affects postoperative clinical outcomes and ventilatory function recovery in LT recipients. In this study, 32.4% of all LT recipients had a preoperative DD, and 54.5% showed gradual improvement after LT. Preoperative DD was associated with a longer postoperative stay in the ICU and longer durations of ventilator use. Patients also showed higher ventilatory inhomogeneity, lower lung volume, and delayed ventilatory function recovery during the early periods. Interestingly, preoperative DD led to a higher prevalence of postoperative DD, and it persisted in 15% (5/33) of patients after 1 year. Therefore, evaluation of diaphragmatic function should be considered in the preoperative assessment for LT, and careful follow-up of diaphragm function is required in patients with preoperative DD.

Several studies have reported that postoperative DD affects clinical outcomes after LT. Postoperative DD increases postoperative morbidity and delayed pulmonary functional recovery, and eventually affects patients’ quality of life after LT [10, 20–22]. However, attention in preoperative DD is still lacking. This study demonstrates the natural course of changes in diaphragm function after transplantation according to preoperative DD. Approximately half of the patients with preoperative DD had restored diaphragmatic function within 3 months after LT, and the remaining 30.3% of patients recovered after 1 year [23]. About half (*n* = 7) of the patients with sustained preoperative DD at 3 months died, 6 patients died from pneumonia and sepsis, and 1 patient died from dehiscence of bronchial anastomosis (Table 4). The median duration of mechanical ventilation after transplantation in 7 patients was 31 days. Sustained diaphragm dysfunction after transplantation may cause prolonged ventilator use, which can lead pneumonia and death. Newly developed DD after LT caused by phrenic nerve injury was temporary in 5.9% of 102 cases. Although direct comparison is difficult due to the nature of the retrospective study, the incidence was comparable with that reported in some previous studies [20, 21, 24], but lower than that reported by Crothers et al. [22]. Finally, persistent DD was observed in only seven patients (6.8%) after 1 year.

In this study, preoperative DD was significantly associated with prolonged mechanical ventilation use after transplant. To understand the background of these associations, we evaluated the difference in ventilation function and functional volume status between the two groups using EIT and 3D-CT volumetry. Patients with preoperative DD showed more inhomogeneous ventilation functions after transplantation [25, 26]. Impaired function of the diaphragm always affects ventilatory function, because the diaphragm is a major inspiratory muscle. Even unilateral DD can affect ventilation asymmetry, and inhomogeneity in lung ventilation leads to impairment of gas exchange, which makes weaning from the ventilator difficult. Moreover, preoperative DD significantly affected total lung volume and functional capacity. The serial improvement in total lung volume also tended to be lower in the preoperative DD group. These findings suggest that preoperative DD not only has a negative impact on early clinical outcomes but also jeopardizes pulmonary function for a long period of time, even in survivors.

This study has several limitations, including its retrospective nature. The participants were mostly in severe clinical conditions requiring mechanical ventilation and/or extracorporeal membrane oxygenation, such that the proportion of preoperative DD might be higher than that in the other population. In addition, the sample size was small to conduct multivariate regression analysis. The clinical outcomes, including pulmonary function and mortality, may be worse than those determined in a previous study [10]. Finally, CT, EIT and ultrasonography were performed at different timepoints, according to the retrospective nature of this investigation. The test results may have affected by the mechanical ventilation in patients with mechanical ventilator. Despite these limitations, the strength of the study included its multidisciplinary approach that provided a novel aspect of the impact of preoperative DD on clinical outcomes and ventilation function after transplantation. In particular, ultrasound is a portable and radiation-free assessment tool that can be performed in all centers for visualizing the diaphragm before transplantation, regardless of the patient's condition. The recognition of such preoperative DD could lead clinicians to conduct proper diagnostic, therapeutic, and rehabilitation treatments, especially in priority patients. Further prospective studies are required to determine the reversibility of preoperative DD.

## Conclusions

Patients with preoperative DD showed poor postoperative outcomes, ventilation inhomogeneity, lower lung volume, and delayed lung function recovery compared with those without DD. Therefore, evaluation of diaphragmatic function should be considered part of the preoperative assessment for LT, and meticulous post-transplantation management is required in patients with preoperative DD.

## Supplementary Information

**Additional file 1. Supplement 1.** Assessment of diaphragmatic function by ultrasonography. **Supplement 2.** Electrical impedance tomography measurements. **Supplement 3.** Diaphragm function changes at 3 months of transplantation.

## Data Availability

The data that support the findings of this study are available from the corresponding author upon reasonable request.

## Abbreviations

DD: Diaphragm dysfunction
DTe: Diaphragm thickness at the end of expiration
DTi: Diaphragm thickness at the end of inspiration
EIT: Electrical impedance tomography
FVC: Forced vital capacity
GI: Global inhomogeneity
ICU: Intensive care unit
LT: Lung transplantation
TFdi: Thickening fraction of the diaphragm
3D-CT: Three-dimensional computed tomography
