# Supplementary material for: Clinical impact of preoperative diaphragm dysfunction on early outcomes and ventilation function in lung transplant: a single-center retrospective study
Source: J Intensive Care. 2022 May 15;10:23. doi: 10.1186/s40560-022-00614-7 (PMC9107696; doi:10.1186/s40560-022-00614-7)
Supplement: Supplementary file 1 — Additional file 1. Supplement 1. Assessment of diaphragmatic function by ultrasonography. Supplement 2. Electrical impedance tomography measurements. Supplement 3. Diaphragm function changes at 3 months of transplantation. [file 40560_2022_614_MOESM1_ESM.docx]

Supplement 1. Assessment of diaphragmatic function by ultrasonography

The M-mode was selected for right diaphragm excursion in the midclavicular line in the subcostal area to visualize the diaphragm dome. The left diaphragm was visualized using the spleen as an acoustic window. The angle of the ultrasound was not less than 70° when aiming for the hemidiaphragm domes. Diaphragm excursion was defined as the length of the vertical axis movement from the baseline to the point of maximum height of inspiration. The excursion of the diaphragm was measured during both tidal and forced breathing. The B-mode was conducted to measure thickness in the anterior axillary line in the apposition zone, where we could visualize the diaphragm peeling away during inspiration. The thickness of the diaphragm was measured at the end of inspiration (DTi) and at the end of expiration (DTe). The thickening fraction of the diaphragm (TFdi) was calculated using the following formula: TFdi = $\frac{(DTi-DTe)}{DTe}$. High-resolution scans were obtained using the LOGIQ E9 ultrasound system (GE Healthcare, Milwaukee, WI, USA). Measurements were taken during 10 breaths and repeated three times for each hemidiaphragm. The data were averaged, excluding those for the smallest and deepest breaths.

Supplement 2. Electrical impedance tomography measurements

EIT measurements were performed postoperatively on day 7 using the PulmoVista 500 (Dräger Medical GmbH, Lübeck, Germany). An array of 16 electrodes was placed around the chest wall in the sixth intercostal space. Ventilation distribution between the right and left lungs at both the upper and lower lung segments during tidal breathing was assessed. To quantify the tidal volume distribution, the EIT-based global inhomogeneity (GI) index was calculated, which represents an asymmetrical distribution in the lungs. The calculation of the GI index has been described in several previous studies^17^. The median impedance value between two selected points in time within the lung regions and the sum of the absolute difference between the median value and each pixel value were considered as indications of variation in the ventilation distribution. The GI index was calculated as follows:

GI = $\frac{\Sigma x,y lung [DIxy-Median (DIlung)])}{\Sigma x,y lungDIxy}$

To reduce the influence of fluid overload and pleural effusion on [EIT](https://www.sciencedirect.com/topics/medicine-and-dentistry/electrical-impedance-tomography), we excluded patients with [pulmonary congestion](https://www.sciencedirect.com/topics/medicine-and-dentistry/lung-congestion) and pleural effusion detected on ultrasonography.

Supplement 3. Diaphragm function changes at 3 months of transplantation

|  | Pre-operative DD (n=33) | Without pre-operative DD (n=69) | P |
| --- | --- | --- | --- |
| DD at 3 months, N (%) | 15 (45.5) | 6 (8.7) | <0.001 |
| Unilateral DD at 3 months, N (%) | 9 (27.3) | 3 (5.4) | 0.003 |
| Diaphragm movement at 3 month, N (%) |  |  |  |
| Resting state, cm |  |  |  |
| Rt | 1.9 [0.8-2.6] | 2.3 [1.7-3.0] | 0.069 |
| Lt | 1.5 [0.5-3.0] | 2.6 [2.0-3.2] | 0.036 |
| Forced state, cm |  |  |  |
| Rt | 4.5 [2.5-5.8] | 4.9 [3.6-6.2] | 0.478 |
| Lt | 3.4 [1.3-5.9] | 5.5 [3.8-7.1] | 0.033 |
| Thickness fraction Rt (%) | 75.5 [19.5-117] | 100 [31-152] | 0.632 |
| Thickness fraction lt (%) | 76 [52.8-121.5] | 111.5 [51.5-139.8] | 0.093 |

Data are presented as medians (interquartile range) or as numbers (percentage).

DD, diaphragm dysfunction; Rt, right; Lt, left.
